# Pre‐adolescent children's experiences of receiving diabetes‐related support from friends and peers: A qualitative study

**DOI:** 10.1111/hex.12802

**Published:** 2018-07-02

**Authors:** David Rankin, Jeni Harden, Katharine D. Barnard, John Stephen, Simita Kumar, Julia Lawton

**Affiliations:** ^1^ The Usher Institute of Population Health Sciences and Informatics University of Edinburgh Edinburgh UK; ^2^ Human Development and Health University of Southampton Southampton UK; ^3^ Borders General Hospital Melrose UK; ^4^ Children's Health Scotland Edinburgh UK

**Keywords:** children, pre‐adolescents, qualitative research, self‐management, type 1 diabetes

## Abstract

**Background:**

While pre‐adolescent children with type 1 diabetes receive most support from their parents/caregivers, others also contribute to their care. This study explored pre‐adolescent children's experiences of receiving diabetes‐related support from friends and peers. The objective was to identify how children could be better supported by their friends and peers to undertake diabetes self‐management.

**Methods:**

In‐depth interviews with 24 children (aged 9‐12 years) with type 1 diabetes. Data were analysed using an inductive, thematic approach.

**Results:**

Children gave mixed accounts of their experiences of speaking to their school/class about diabetes with some indicating that this had resulted in unwanted attention. Most individuals reported that other children had a limited understanding of diabetes and sometimes acted in insensitive ways or said things they found upsetting. Virtually all children described having a small number of close friends who were interested in learning about diabetes and provided them with support. These friends provided support in three overlapping ways, as “monitors and prompters,” “helpers” and “normalizers.” While some children described benefiting from meeting peers with type 1 diabetes, most indicated that they would prefer to develop friendships based on shared interests rather than a common disease status.

**Discussion and conclusions:**

Friends and peers provide several kinds of support to pre‐adolescent children with diabetes. Health professionals could consider ways to assist small friendship groups to undertake monitoring and prompting, helping and normalizing roles. Parents, schools and health professionals could explore ways to normalize self‐management practices to better support children with diabetes in school settings.

## 1 INTRODUCTION

Type 1 diabetes is one of the most prevalent chronic conditions that develops in childhood.^1^ Self‐management is a complex and demanding task requiring: frequent monitoring of blood glucose (at least 4 times daily), injecting (at least 4 times a day) or inserting insulin pump infusion sets (every 2‐3 days); close monitoring of food intake and counting carbohydrates; calculating insulin doses; regulating physical activity; and monitoring and treating hypoglycaemia and hyperglycaemia.^2^ While children are encouraged to participate in these diabetes management tasks from an early age, young children often lack the skills necessary to undertake self‐management independently and parental/caregiver involvement remain essential throughout childhood.^3^

Although parents/caregivers provide the majority of diabetes‐related support, other individuals are also involved, including friends and peers.^4^ As a recent synthesis of qualitative studies exploring the everyday experiences of pre‐adolescent children with type 1 diabetes has highlighted,^4^ friends provide several kinds of support, including enquiring about their well‐being^5^, ^6^; obtaining hypoglycaemia treatments^6^, ^7^, ^8^; and making adjustments to their own lives to accommodate dietary and lifestyle restrictions.^6^ However, most of the studies included in this synthesis explored children's broader experiences of managing diabetes in everyday life,^5^, ^7^, ^8^, ^9^ with only one focusing on how friends provide support.^6^ While offering important insights, this study was undertaken in Brazil; hence, the findings may not be generalizable to other contexts.

To supplement the limited qualitative research undertaken to date, we conducted an interview study with children aged 9‐12 years with type 1 diabetes. A key aim was to explore their experiences of receiving diabetes support from friends and peers and whether, how and why they found this support helpful. Our objective was to identify ways in which pre‐adolescent children can be better supported by friends and peers to undertake diabetes self‐management. The decision to focus on children aged 9‐12 years was made by the study advisory group comprising clinicians, diabetes experts and academics with expertise in family and child health. In keeping with previous research,^10^ this group identified the pre‐adolescent years as a critical time during which children take on more responsibility for managing their diabetes as well as developing closer relationships with peers.^11^

## 2 METHODS

### 2.1 Study design

The study comprised in‐depth interviews using age‐appropriate questioning, incorporating optional play‐based tasks, to take account of children's differing ages and capabilities^12^ and enable them to discuss issues they perceived as salient, including those unanticipated at the study's outset.^13^ An inductive approach was used which entailed simultaneous data collection and analysis with findings identified in early interviews iteratively informing areas explored in subsequent accounts.^14^ Recruitment and interviewing continued until data saturation occurred; that is, until no new findings were identified in new data collected.

### 2.2 Recruitment and sample

Children (aged 9‐12 years) were recruited from four Scottish paediatric diabetes centres, spanning rural and urban locations. After obtaining parents’ permission, health professionals approached children during routine clinical consultations using an opt‐in procedure. Purposive sampling was used to ensure there was diversity in children's age, gender, and diabetes duration. The sample comprised approximately equal numbers of children using multiple daily injection or pump regimens, in line with usage by this age group across Scotland. Children had to have been diagnosed for at least 6 months to ensure that they had had sufficient time to make physical and psychological adjustments to having type 1 diabetes and to establish an intensive insulin regimen. All participants and their parents/carers completed written, age‐appropriate consent forms.

### 2.3 Data collection

Interviews were conducted by DR, an experienced qualitative researcher, who had attended professional training on ways to involve young children in research. Interviews were informed by a topic guide developed using literature reviews, inputs from advisory group members and revised in the light of emerging findings. Relevant areas explored during interviews are shown in Table 1. Interviews took place between July 2016 and February 2017 in children's homes, with one‐third of children choosing to be interviewed with a parent/carer present. Interviews averaged 45 minutes were digitally recorded and transcribed in full.

**Table 1:** Relevant areas explored in interview topic guides

What sort of things do children do with their friends (e.g. sports, playing, hanging out)? What do children's friends know about type 1 diabetes? What have children told their friends about type 1 diabetes? How does having diabetes affect the things children do with their friends? Do children get treated any differently by their friends because they have diabetes? What are children's experiences of being provided with diabetes‐related support by their friends? What sort of things do friends do to help? Do children know other people who have type 1 diabetes, including other children? How did they meet these individuals, and was it helpful to speak with them about diabetes? What sort of things do children talk about with their peers? Would children find it helpful to speak to other children who have diabetes? Why/why not?

### 2.4 Data analysis

A thematic analysis using the method of constant comparison^15^ was undertaken by two experienced qualitative researchers (DR and JL), who were not part of a clinical team. Participant interviews were read through repeatedly before being compared to identify cross‐cutting themes. Regular meetings were held to compare interpretations, reach agreement on recurrent themes and findings and agree on a coding framework which captured original research questions and emerging findings. All data were coded using NVivo, a qualitative software package (QSR International, Doncaster, Australia). Coded datasets were subjected to further in‐depth analyses to allow more nuanced interpretations of the data and to identify illustrative quotations.

Ethical approval was provided by the South East Scotland Research Ethics Committee 01, NHS Lothian (16‐SS‐0084). Below, data are tagged using unique identifiers.

## 3 RESULTS

The sample comprised 24 children (see Table 2 for further details). No apparent differences were noted in the characteristics or responses of children who were interviewed with or without parents/carers present. We begin by briefly reporting children's experiences of informing and educating friends and peers about diabetes. Next, we report children's accounts of friends/peers who had been unsupportive, or had acted insensitively, when they were undertaking diabetes‐related tasks, and the upset and distress this had caused. Using children's accounts, we illustrate how friends had engaged with, and learned about, diabetes and had provided three overlapping types of support by acting as “monitors and prompters,” “helpers” and “normalizers.” We conclude by reporting children's experiences of, and views about, receiving peer support from other children with type 1 diabetes.

**Table 2:** Demographic characteristics of interview participants

Characteristic	N	%	Mean ± SD & range
Children (n = 24)			
Female	11	45.8	
Age—all children			10.3 ± 0.9, range 9‐12
Female age at time of interview (y)			10.4 ± 1.1, range 9‐12
Male age at time of interview (y)			10.2 ± 0.8, range 9‐11
Female age at diagnosis (y)			5.0 ± 3.0, range 1‐10
Male age at diagnosis (y)			6.7 ± 2.1, range 3‐10
Diabetes duration—all children (years since diagnosis)			4.3 ± 2.4, range 1‐10
Regimen (at time of interview)			
Basal‐Bolus	11	45.8	
CSII (insulin pump)	13	54.2	

### 3.1 Informing other children about diabetes

Many children described having given a talk or presentation to their classmates to explain what diabetes is and how the disease is managed. While several children said it had been helpful to speak or present to a large group, others noted how many of their classmates had struggled to understand the contents of their presentation:

I guess they know a little bit… cause I did the PowerPoint [presentation] and then they were asking me so many questions, like they don't get the PowerPoint at all. … So I don't think they know––I think they know a teeny bit (002).

Some children also highlighted how giving a presentation had resulted in them receiving unwanted attention from classmates and being asked repetitive questions: “they were just constantly on my back… they were just always asking” (013). Indeed, to minimize the likelihood of being asked intrusive questions, 007 reported choosing not to disclose her diabetes to her class and to speak only with trusted confidantes:

I don't really need the whole one thousand, like however many kids there are, knowing and like asking questions and stuff. So it's sort of just like my friends who need to know.

### 3.2 Insensitive and unsupportive peers

Children also reported how many of their friends and peers had little or no understanding of what they needed to do to manage diabetes and how, as a consequence, they sometimes acted in insensitive ways or said things which were upsetting. This included several children who described how their classmates did not understand why they needed to eat sugary foods in lessons in order to treat hypoglycaemia; such misunderstandings, as 001 indicated, could leave children feeling vulnerable to teasing and resentment:

there's this kid in my class… He's quite a‐ like, I'd say rude. I'm like‐ he thinks he's so perfect. […] eh, he's always just been like: ‘how does he get to eat [sugary food]?’ And then just complains to the teacher. And no matter how many times I tell him he just doesn't understand.

Children also talked about occasions when friends or peers had expressed doubts about their ability to participate in activities such as playground games or sports because of the risk posed by hypoglycaemia. 011, for instance, described feeling very upset when some of his friends had suggested that he should be excluded from a game of rugby:

they said like, ‘oh, if you're gonna run about, what if you go low? I think maybe you should stay inside or something’. And I thought, ‘nah, I'm gonna be fine’. […] I feel like sometimes it can be annoying, cause I know what's right. But they don't…

As well as having to cope with unkind comments and exclusion from play activities, several children provided examples of friends and peers acting insensitively when they left lessons early to check their blood glucose levels and administer insulin before lunch. This included 010 who reported feeling isolated and cut off from his peers because no one would wait for him before eating their lunch:

I think everyone ends up eating before me, cause I like get to leave [class] early before them. But then they go for lunch and whilst I'm getting my jags [injections] and blood and stuff [and] they're normally like on their like second last thing of their lunch. So I don't get the chat or anything.

Further insensitivities were discussed by children who reported feeling upset when their friends shared sweets with each other which they were not permitted to eat: “it's annoying, cause people like bring sweets into school and they share them out, and I can't have any” (003). Recalling similar experiences, 023 described having chosen to withdraw from his classmates on such occasions “so I don't get tempted” and to mitigate feeling upset:

I'll just go to a different room so I don't get tempted. It just makes you feel horrible and I get hungry when that happens. […] I won't look at them. I just won't look at the sweets, but I'll talk to them. Just forget about it. And my mouth waters when I look at them.

In a related example, 007 described feeling very upset when she had attended a classmate's birthday party where other children had ready access to sugary foods and drinks which she was not allowed to consume. This experience, as 007 went on to report, had left her feeling socially isolated and desperate to leave the party:

I remember, I just had this drink, and it was like some sort of diluted drink that had no sugar in it. And everyone else was drinking like full fat Coke and eating a bunch of food. And I was just sitting there like, one biscuit… I sort of wanted to just phone my mum and say, ‘just pick me up. Sorry, I don't want to be here anymore.’

### 3.3 Support provided by friends

#### 3.3.1 Friends’ development of knowledge about diabetes

While all children shared the kinds of negative experiences reported above, most also described having a small number of friends who wanted to be supportive and expressed an interest in learning about their diabetes. This included 002 who described how: “my friends are curious about diabetes so I tell them like about how I feel high or low and stuff like that” and 007 who, after her best friend had expressed an interest in learning more, reported having sat down with her to read a book designed to help children better understand diabetes:

she [best friend] probably saw me before even like most of my family found out. … And then like I think we got this wee small book that was like explaining it for children. And like my mum and me like sort of explained it [diabetes] to her.

Some, including 003, described how they had capitalized upon their friends’ interest and curiosity by inviting them to watch when they undertook tasks, such as administering injections, and then answering their questions:

my friends are really supportive. Em, they know a lot about it … because when I was doing my injections I took them down to the medical room. They saw, and they asked questions and I answered them and they just learned about it.

#### 3.3.2 Monitors and prompters

As many children reported, friends who wanted to help and better understood diabetes often provided them with welcomed and valuable support. In several cases, children described how their friends had acted as monitors by identifying changes in their bodily state or behaviours indicative of hypoglycaemia. For example, 005 reported that “one of my good friends from school, she really understands it … she knows when I'm hypo and stuff. She can just kind of see that I'm going a bit dizzy and pale and shaky.” Other children also described how friends who took on this monitoring role often prompted them to check their blood glucose. This included 013 who reported how her friends had helped on occasions when she struggled to detect low blood glucose:

there's been a few times where I kinda act like different than what I normally would. I kinda loose concentration and whatever if I'm having a hypo and whatever. And if they [friends] recognise that, they'd be like: ‘maybe you need to check your glucose or whatever.’

Children also illustrated how friends who acted as monitors had sometimes pre‐empted and alerted them to potential threats by raising concerns in a sensitive manner which invited personal reflection: “sometimes I look like I'm low he asks like, ‘are you okay with your blood sugars?’” (017). In another example, 008 described how his friend had gently prompted him to consider the risk of developing hypoglycaemia in advance of undertaking physical activity: “the friend I was talking about, he's more worried in terms of activities, like him asking if I'm sure I'll be able to do this [activity] due to my blood sugars” (008). Similarly, as 024 described, his best friend was aware that he should limit his intake of sweets and would, if necessary, subtly prompt him to reconsider whether he should consume sugary foods out of concern for his well‐being:

he looks out for me when it comes to my sugar, like what my sugar's like and, and if I say, ‘can I get one of them [sweets]?’, he says, ‘would you be allowed it?’ And he says things like that, technically looking out for me, so I don't like die or go into a coma.

In other cases, children described how close friends often became very aware of their diabetes‐related routines and, at specific times, would prompt them to undertake tasks such as blood glucose self‐monitoring or administering insulin. Specifically, children recounted how their friends had reminded them if they forgot to perform such tasks, including on occasions when they became so engrossed in activities that they lost awareness of time:

he [friend] like tells me sometimes when to take my bloods, cause I forget sometimes. He says, ‘you'll need to take your blood now’. And then when I eat something accidentally without putting carbs in, he's like, ‘I think you need to put carbs in [to a pump to determine an insulin dose] or something’. So he reminds me. It's just that I do it at school so much, that he knows I do it (004).

they [friends] would remind me, if I'm too busy playing the game, like, ‘oh, it's quarter to, go and get your jag [injection]’ and stuff like that (022).

#### 3.3.3 Helpers

Children also described how their friends had provided practical support by assisting them to perform diabetes management tasks. This included some who reported how their friends had helped at school when they needed to treat hypoglycaemia by retrieving treatments from storage: “if I am kinda like dizzy and whatever, and if I kinda just needed to sit down, I could ask them to get my Lucozade and they would get it” (013). Similarly, other children described how their friends had accompanied them to the medical room at school to ensure they were able to safely retrieve treatment for hypoglycaemia:

most of the time when I feel low or that, my friends would always be there for me, saying like, ‘oh, I'll come with you [to fetch treatment]’ and that kind of thing (014).

In other examples, children talked about receiving help from friends when the debilitating effects of hypoglycaemia had affected their ability to respond to and treat low blood glucose. This included 005 who described how her friends had offered encouragement when she had felt too confused and disoriented to respond then treat hypoglycaemia: “they understand if I'm hypo, I can't just do it. And if I'm dithering over doing a [finger prick test], they just say, ‘just do it’” (005). Others illustrated how their friends had offered practical support by alerting adult caregivers when they needed assistance to manage hypoglycaemia: “he would call my mum while I do my blood and do my dextrose, and say that I'm in a hypo. And then my mum would come down [to collect me]” (006).

#### 3.3.4 Normalizers

As well as providing practical assistance, children discussed how their friends acted in ways which helped normalize their experience of having, and managing, diabetes. Specifically, children reported how friends had made adaptations to their own lives so they did not need to compromise self‐management activities in order to fit in with their peers. Indeed, when friends acted in this way, children discussed being able to remain connected to, and embedded within, their social networks and thereby feel “normal.” This included 019 who recounted how one of her friends delayed having lunch at school until she had completed self‐management tasks so they could eat together:

…at lunchtime today she waited for me to like do my blood sugar and to put my insulin in, then show my teacher. She waited for me. And sometimes she just waits on me and stays with me before we eat.

Children also illustrated how friends had helped to normalize their experiences if they needed to interrupt play activities or meal times in order to treat and recover from an episode of hypoglycaemia. 004, for instance, described how his best friend, unlike other classmates, would wait for him to recover: “if I'm like hypo he stops and he sits down with me. And he waits for me and everything. Like cause somebody else would just run about and forget about me.” Similarly, 005 recalled an occasion when she had attended a sleepover where her friends had waited for her to treat and recover from an episode of hypoglycaemia before continuing to eat together:

I was hypo em when everyone was having pizza at my friend's, we were all having a sleepover … [It's] very important actually, because I don't like, like being like left behind, like just cause I've had a hypo, I'm a bit behind or something. And if everyone's finished their pizza and then I have to have it and they're maybe off, doing something, playing a game. And then I come through and they're like halfway through that game … but they waited, and it's good.

Others described occasions when friends had provided support by adapting their eating behaviours and food choices to accommodate and normalize the dietary restrictions they faced. For example, 007 discussed how, while playing at another child's house, her friend chose only to eat foods which both girls could consume and delayed the time of a snack to ensure that both ate at the same time:

if we're like at her house, she won't like eat something just randomly. She'll always ask if I can have it as well, like if it's okay. [And], if we've just had lunch, and like an hour later she's hungry, her mum and her'll just like wait until, you know, that I'm like allowed something, and then we'll both have the same thing.

### 3.4 Peer support provided by other children with type 1 diabetes

While all children talked about benefiting from support provided by select friends, many recalled mixed experiences of meeting other children with type 1 diabetes at organized groups/events. Others also reported feeling ambivalent about whether they might benefit from speaking with, and seeking support from, peers in future. Indeed, although some children speculated that speaking to peers with diabetes might help reduce social isolation: “so that I don't feel like I'm just the only person that thinks they have diabetes” (023), several cast doubt on whether such encounters would lead to lasting and supportive relationships. This included 007 who reported that she would prefer to develop friendships of her own volition rather than as a result of being brought together as individuals, or in groups, on the basis of a shared disease status:

I sort of think if I was to meet someone… like it would be okay. It wouldn't really help me lots. Like if I was to meet someone, just out of coincidence, I would think that's better than like going to a group … then that's like having made my own friendship … so if I made a friend who happened to have it, I might end up talking to them about it, but probably not that much. It might come up every now and then. And we would be able to relate to each other. But not so much that I would intentionally seek it out for a person to have diabetes that's a friend.

Indeed, several individuals who had attended organized diabetes support groups described having not formed friendships with other children because, as 003 indicated, they did not want to be defined by, or feel that they needed to talk about, having diabetes:

I know a lot of children [with type 1 diabetes] cause I go to the support group. But I'm not really good friends with any of them. […] it's good cause everyone has the same thing and the same problem… and I don't mind talking about too much, but I don't like talking about it all the time.

In related examples, several children reported having struggled to find common ground on occasions when they had met or been introduced to peers with diabetes. This included 001 who described how he had found it difficult to relate to two younger children with diabetes at his school because: “they're 2 years younger than the P7's [final year of primary school]… I mean, they're so different and they think about things differently too.” Indeed, as several other children indicated, when they had been introduced to and become friends with peers who had diabetes, their friendship and the types of things they talked about were mainly based on having shared interests, rather than conversations about diabetes. This included 014 who reported having become friends with another child with diabetes because they had a shared interest in football:

we don't really need to [talk about diabetes], cause like we just can talk about like football and like what we're meant to be doing… Like there's other things that we could talk about together. Like more interesting things and that.

A few children did, however, discuss benefiting from meeting peers with diabetes in group settings. This included 016 who described picking up “handy tricks” from his peers at a diabetes camp he attended soon after starting to use an insulin pump: “cause this pump, I had only had it not that long. So they [other children] taught me quite a lot of handy tricks to use, how to keep your blood low.” Others, however, speculated that they would be reluctant to take on new self‐management practices suggested by peers with diabetes because, as 013 explained, she had developed her own unique approach to performing diabetes‐related tasks: “I don't think it would help [to meet others] because obviously everyone with type 1 diabetes is different, like everyone has different ways of controlling it and like varied blood glucose.” Likewise, other children expressed doubt about whether they would benefit from diabetes‐specific suggestions made by peers because, as 008 reported, their self‐management practices were informed and guided by health‐care professionals and their parents:

I'm not sure [about meeting other children], because whether or not they do say something of note, it's down to my mum as to whether I can change onto that or not. Em, so it seems that doctors and nurses tend to know more about it than people themselves, in terms of a technical level.

## 4 DISCUSSION

This is one of few studies to explore pre‐adolescent children's experiences of seeking and receiving support from friends and peers to help manage type 1 diabetes. Participants described how many of those within their wider peer group struggled to understand diabetes and appeared to act insensitively, or in ignorance. This included individuals who questioned their ability to take part in activities or eat certain foods, and being unsupportive when they performed diabetes‐related tasks, such as checking blood glucose and administering insulin. Children also highlighted the upset, distress and isolation they experienced as a consequence. For this reason, and in keeping with findings reported in other studies,^4^, ^6^ children described preferring to interact with, and accept support from, a small number of friends who wanted to help and were interested in learning about diabetes. These friends, as children reported, had often developed knowledge about diabetes by asking questions opportunistically and when observing them perform self‐management tasks, which then enabled them to provide three overlapping types of support as “monitors and prompters,” “helpers” and “normalizers.” While valuing this practical and emotional input from selected friends, children reported more mixed and ambivalent views about receiving support from peers with type 1 diabetes.

As our findings suggest, children reported only limited benefit from speaking to peers who have type 1 diabetes in order to reduce feelings of isolation and obtain advice about diabetes‐related tasks. This, as many children suggested, was because they preferred to engage with other children on the basis of having similar interests (e.g. playing football) rather than a shared disease status. For this reason, it could be argued that our study provides only caveated support for models of peer support which bring children with type 1 diabetes together, such as at diabetes camps^16^ or in group sessions,^17^ and highlights a need for other day‐to‐day peer support approaches to be considered. Given that close, self‐selected friends both want and are well situated to give the kinds of emotional and practical support which pre‐adolescent children value, we suggest that one such approach would be to work closely with small friendship groups to provide the education and support they need to undertake safe and effective monitoring and prompting, helping and normalizing roles. To achieve this, further research may need to be done with close friends to establish what their information and support needs are. It may also be necessary to work closely with schools and parents to help ensure they can enable friendship groups to provide support to the child with type 1 diabetes. In the case of schools, this might include offering flexible canteen arrangements so children with diabetes can eat together with their friends. These recommendations might also be applicable to older age groups such as adolescents with type 1 diabetes. This is because a synthesis of studies undertaken with adolescents has similarly highlighted how friends can both help and hinder diabetes self‐management.^18^

We have also highlighted how children can feel distressed when they encounter peers who, because they do not understand diabetes, can act in seemingly insensitive, inappropriate and unsupportive ways. Other studies have shown that children with diabetes can be vulnerable to bulling by friends and peers^4^ and although children in our study did not explicitly state having been bullied, their accounts suggest that this experience cannot be discounted. While some close friends do appear to be capable of understanding diabetes and ways to manage the disease, we would suggest that the problems our participants described are unlikely to be the result of their wider peer group being unable to comprehend and apply diabetes learning. Rather, as some children in our study noted, the didactic approaches they were encouraged to use, where they educated their class or school by delivering a talk, do not appear to be an effective way of engaging and educating classmates. For this reason, we would suggest that schools and health professionals may need to consider alternative ways to educate pre‐adolescent peers. Given that opportunistic and experiential approaches seemed an effective way of engaging and cascading diabetes knowledge to close friends, one strategy might be to allow classmates or friends to observe diabetes‐related tasks being performed, for example an injection being administered, with an explanation given about why this is necessary and the intended outcome. While some studies suggest people with diabetes feel the need to undertake self‐management tasks, such as injecting, in private due to fears about stigmatization, this work has mostly been conducted with adults who fear others will associate injecting with drug use^19^, ^20^; hence, this is less likely to be an issue or concern for pre‐adolescent children. We also suggest that, if diabetes self‐management activities such as checking blood glucose and injecting are taken out of concealed environments such as medical rooms, this might help demystify the condition and, hence, potentially, reduce instances of teasing. This recommendation is in keeping with findings reported by the authors of a recent synthesis of studies exploring how intensive insulin therapy is used in primary schools^21^ who have suggested that to “create a sense of normality and understanding of diabetes at school, it is important to advocate for a wellness approach, reduce stigma, address fear and provide collaborative support.” As the authors further note, segregating diabetes care away from usual school activities may bolster and reinforce negative attitudes from school staff and peers as this suggests that diabetes is an illness, rather than part of normal life. Hence, they recommend collaboration between home, health, education and legal systems to explore integration of care into usual school routines to enhance normality, understanding and a wellness approach. Our recommendations might also be used to enhance the support provided by friends of children with other chronic conditions. Similar to the children in our study, recent reviews have found that children with asthma and epilepsy report benefiting from emotional and practical support provided by close friends. These studies have also highlighted a need to educate wider friendship groups in order to help normalize children's experiences of self‐managing their disease.^22^, ^23^

A key question which this study, like the synthesis by Marks et al,^21^ raises, is whether the difficulties encountered by pre‐adolescent children with type 1 diabetes might be avoidable through changes in culture and practices within schools and other settings. A particularly emotive issue raised by the children in our study was the upset they experienced when they were unable to eat the same foods and snacks as classmates, and the sense of isolation and alienation they reported as a consequence. As well as helping to raise awareness and understanding of the distress which children may encounter, it should be noted that, with the widespread use of flexible intensive insulin regimens involving multiple daily injections or insulin pump therapy,^24^ it should be possible for young people to eat the same types of foods and snacks as their peers as long as an appropriate insulin dose is administered. To achieve this, greater awareness, understanding and resourcing may be required within schools so that more staff are empowered and upskilled to offer appropriate diabetes management support. Better education may also need to be provided to parents to increase their confidence to use flexible intensive insulin regimens^24^ and in order to support these being used when children are at school. Indeed, these may be vital steps to help address feelings of low self‐esteem which can affect social development and disease mismanagement in children with type 1 diabetes.^25^, ^26^

A fundamental study strength is our use of an exploratory, open‐ended design as this has allowed new and unanticipated issues to be identified, namely the different types of support provided by friends and children's ambivalence about receiving support from peers with type 1 diabetes. Using this design, we have enhanced understanding of the difficulties and distress young people with type 1 diabetes may encounter, as well as the vital role which small friendship groups may play in countering this distress and helping them to undertake effective diabetes self‐management. A potential limitation is that children may have used their interviews as an opportunity to present themselves in a positive light.^27^ Furthermore, we did not include the perspectives and experiences of close friends and other peers; hence, our recommendation that future research be conducted with these individuals. An additional limitation is the restriction of our study to one geographical setting, Scotland, where practices within schools may be different to those in other countries, albeit as the review by Marks et al suggests, the sequestering of activities such as blood glucose monitoring and injecting in offices and medical rooms in school, may be commonplace.^28^

## CONFLICT OF INTERESTS

DR, JH, KB, JS, SK and JL have no relevant conflict of interests to disclose.
